# Effects of uric acid-lowering therapy (ULT) on renal outcomes in CKD patients with asymptomatic hyperuricemia: a systematic review and meta-analysis

**DOI:** 10.1186/s12882-024-03491-4

**Published:** 2024-02-23

**Authors:** Yuxin Luo, Qirong Song, Jiaxiao Li, Sha Fu, Wenjuan Yu, Xiaofei Shao, Jinxiang Li, Yuliang Huang, Junzhe Chen, Ying Tang

**Affiliations:** Department of Nephrology, The Third Affiliated Hospital of Southern Medical University, Guangzhou, China

**Keywords:** Hyperuricemia, Renal Insufficiency, Chronic, Uric Acid, Renal Dialysis, Gout

## Abstract

**Background:**

It is well known that asymptomatic hyperuricemia and gout play an important role in patients with chronic kidney disease (CKD). However, the effect of uric acid-lowering therapy (ULT) on the prognosis of CKD patients with asymptomatic hyperuricemia remains controversial. Therefore, we aim to investigate the influence of ULT on renal outcomes in these patients.

**Methods:**

Comprehensive searches were conducted in PubMed, EMBASE, China National Knowledge Internet (CNKI), and the Cochrane Library, up until January 2024. We included randomized controlled trials (RCTs) that evaluated the effects of ULT on renal outcomes in CKD patients with asymptomatic hyperuricemia.

**Results:**

A total of 17 studies were included in the meta-analysis. Compared with placebo or no treatment, ULT preserved the loss of estimated glomerular filtrating rate (eGFR) (Weighted mean difference [WMD] and its 95% confidence intercal(CI): 2.07 [0.15,3.98] mL/min/1.73m^2^) at long-term subgroup. At the same time, short-term subgroup also proved the preserved loss of eGFR (WMD 5.74[2.09, 9.39] mL/min/1.73m^2^). Compared with placebo or no treatment, ULT also reduced the increase in serum creatinine (Scr) at short-term (WMD -44.48[-84.03,-4.92]μmol/L) subgroup and long-term (WMD -46.13[-65.64,-26.62]μmol/L) subgroup. ULT was associated with lower incidence of the events of doubling of Scr without dialysis (relative risk (RR) 0.32 [0.21, 0.49], *p* < 0.001). However, no difference was found for lower incidence of acute kidney injury (AKI) (*p* = 0.943).

**Conclusions:**

According to our study, ULT is beneficial for slowing CKD progression both in short to long-term follow-ups. Additionally, in patients younger than 60 years old, the protective effect of ULT on renal outcome is more pronounced. However, it showed no significant difference in the incidence of AKI. These findings underscore the importance of considering ULT in clinical strategies for CKD patients with asymptomatic hyperuricemia.

**Supplementary Information:**

The online version contains supplementary material available at 10.1186/s12882-024-03491-4.

## Introduction

In the past few decades, hyperuricemia and gout have become more prevalent worldwide [1, 2]. A cross-sectional survey of 3,547 chronic kidney disease (CKD) patients found that the prevalence of hyperuricemia in CKD patients with stages 3–5 were 42.6%, 59.1%, and 61.2% respectively in China [3]. Hyperuricemia is associated with not only high risk of gout flare but also the increased risk of renal prognosis and cardiovascular events in CKD patients [4–7]. The development of gout from asymptomatic hyperuricemia is a continuous process [7], starting from asymptomatic hyperuricemia to urate-crystal deposition in joint cavities, and finally gout and its complications [8].

Hyperuricemia has been implicated in the acceleration of CKD. The pathogenesis of uric acid-induced renal damage involves multiple mechanisms. Uric acid-induced endothelial dysfunction, activation of the renin-angiotensin system, decreased nitric oxide production, meanwhile, uric acid-induced inflammation and oxidative stress contribute to glomerular hypertension, hypertrophy, and eventual sclerosis [9, 10].

In previous studies, the effect of uric acid-lowering therapy (ULT) on renal and cardiovascular outcomes are controversial. Some studies showed that febuxostat and allopurinol can reduce uric acid and improve renal function effectively in patients with CKD without clinical symptoms [11–13]. However, other studies did not find out the association [14–17]. Recently, two randomized controlled trials (RCTs) of ULT in CKD patients with asymptomatic hyperuricemia showed that there was no evidence for benefits of ULT on kidney outcomes [14, 18]. The levels of SUA in these RCTs [11–18] were found with broad heterogeneity, patients with prior gout flares and acute gout flares were enrolled and included for analysis.

Based on aforementioned studies, different countries also have distinct suggestions for ULT in CKD patients with asymptomatic hyperuricemia [8, 19–21]. The 2019 Chinese Guidelines for Diagnosis and Treatment of hyperuricemia and gout suggested that CKD with stage 2 or higher should start ULT when the level of SUA exceed 480 μmol/L, which should be maintained below 360 μmol/L. However, current guidelines [8, 20] written by American College of Rheumatology (ACR) [20] and the Gout, Hyperuricemia and Crystal-Associated Disease Network (G-CAN) [8] did not recommend ULT for patients with asymptomatic hyperuricemia, as the effects of ULT in this population has yet to be confirmed.

Therefore, whether ULT should be used in CKD patients with asymptomatic hyperuricemia to prevent the progression of CKD remains uncertain. In this systematic review, we aim to investigate the effects of ULT on renal outcomes in CKD patients with asymptomatic hyperuricemia.

## Methods

### Literature search

We searched RCTs that assessed ULT including febuxostat or allopurinol or other uric acid-lowering drugs versus control group in CKD patients with asymptomatic hyperuricemia through Pubmed, EMBASE, China National Knowledge Internet (CNKI) and the Cochrane Library until January 10,2024. Keywords and relevant terms were used as following: chronic kidney disease, chronic kidney failure, chronic renal insufficiency, chronic renal failure, allopurinol, febuxostat, uric acid-lowing therapy, xanthine oxidase, urate-lowering therapy, Benzbromarone, Probenecid, rasburicase, sulfinpyrazone, lesinurad, topiroxostat. No language restriction was applied. All the review processes followed registered protocol that was accepted by the online PROSPERO international prospective register of systematic reviews of the National Institute for Health Research (https://www.crd.york.ac.uk/PROSPERO/) (CRD42022321527). Definition of PICOS in the present study is as follows: P (Population): CKD patients complicated with asymptomatic hyperuricemia; I(Intervention): uric acid-lowing therapy, such as taking allopurinol or febuxostat and other uric acid-lowering medications; C(Comparison): placebo or usual therapy or no treatment; O (Outcome): the effects of intervention on renal outcomes; S (study design): randomized controlled trial.

### Inclusion and exclusion criteria

Studies that met the following criteria were included in our analysis: (1) Adult CKD patients with hyperuricemia (SUA ≥ 7.0 mg/dl [420.0 μmol/L] in men or ≥ 6.0 mg/dl [360.0 μmol/L] in women) or at least mean baseline SUA ≥ 6.0 mg/dl (360.0 μmol/L) and no prior gout flares; (2) Clearly documented the inclusion and exclusion criteria; (3) Adequately documented the dosage and duration of the intervention and control groups;(4) RCTs.(5) The following outcome measures were used to evaluate the efficacy of agents for hyperuricemia in CKD patients: changes in SUA, changes in Scr, changes in eGFR, acute kidney injury (AKI) or events of doubling of Scr without the requirement of dialysis.

Studies were excluded from our analysis if (1) The baseline data was incomplete, especially devoid of the baseline levels of SUA;(2) Study with less than 20 patients;(3) The outcomes were not clearly documented;(4) Patients with prior gout flares or acute gout flares;(5) reviews, case reports, animal and in vitro experiments, and conference abstracts; (6) Patients with acute kidney injury and those who required dialysis, chemotherapy therapy or had received kidney transplant.

### Data extraction and quality assessment

Two reviewers (LYX and SQR) independently extracted the following information from each included study: first author, year of publication, study population characteristics, study design, inclusion and exclusion criteria, matching criteria, febuxostat or allopurinol or other uric acid-lowering drugs, intervention period, treatment duration, outcomes, and adverse effects. Any disagreement on data extraction was resolved by the third independent reviewer (TY). The authors of the studies were contacted for additional information when necessary.

Risk of bias for each study was assessed by using modified Jadad [22–25] scale and the revised Cochrane risk of bias, version 2 (RoB 2) tool [26]. This tool is comprised of 5 domains addressing biases in the randomization process, deviations from intended interventions, missing outcome data, measurement of the outcome, and selection of the reported result. If a study with all domains rated as 'low risk of bias', it is considered to be of high quality. If one or more domains are rated as 'high risk of bias', the overall validity of the study's findings is questionable and is rated as high certainty of evidence. 'Some concerns' in multiple domains might cumulatively suggest a moderate risk of bias in the study.

### Statistical analysis

All statistical analyses were performed with STATA 14.0 (StataCorp, College Station, TX). The effect size for each study was defined as the weighted mean difference between the treatment group and the control group. If in the original papers results was available only as graphs, the GetData Graph Digitizer (version 2.22, http://www.getdata-graph-digitizer.com) was used to transform them to numeric values. When necessary, standard deviations (SD) were calculated according to the Cochrane Handbook for Systemic Review and Follmann D's method [27]. We have categorized the studies into two subgroups based on their follow-up durations: short-term (3–6 months), and long-term (> 6 months). This approach allows us to minimize the potential impact of varying follow-up times on the outcome measures and enhances the comparability of results across studies. Statistical heterogeneity was assessed using the Chi-square test and the I^2^statistic, with an I^2^value greater than 50% or a *p*-value ≤ 0.05 indicating substantial heterogeneity. An I^2^value between 25 and 50% is considered to represent moderate heterogeneity, while values exceeding 50% denote substantial heterogeneity. We conducted demographic, clinical subgroups analysis in meta-analyses based on characteristics like age, ethnicity, the baseline level of eGFR. Subgroup analyses were conducted to explore sources of heterogeneity under a mixed effects model, which pools studies within a subgroup using a random effects model, but tests for significant differences between subgroups using fixed effects models. The Mantel–Haenszel method will be utilized to determine the effect size of binary outcomes, whereas the inverse variance method will be employed to determine the effect size of continuous outcomes. Sensitivity analysis was performed by eliminating studies one by one and recalculating the pooled effect and eliminating studies with low-quality.What’s more, Egger's test was performed to assess publication bias. When the *p*-value of Egger's test is below 0.05, it suggests statistically significant evidence of publication bias. Conversely, a *p*-value above 0.05 indicates insufficient evidence for such bias.

### Outcome and definition of terms in literature

The primary outcomes included the change in eGFR and Scr from baseline until the end of the study; the secondary outcomes included AKI and doubling of Scr without the requirement of dialysis. Doubling of Scr without the requirement of dialysis is defined as a deterioration of renal function, indicating an increase in Scr values exceeding 100% from the baseline, without requiring dialysis. [28]. The formulas for eGFR in the included literature are mainly the CKD-EPI formula (Chronic Kidney Disease Epidemiology Collaboration) and the MDRD (Modification of Diet in Renal Disease) Study Equation.

## Results

### Study selection and baseline characteristics

**Fig. 1:**
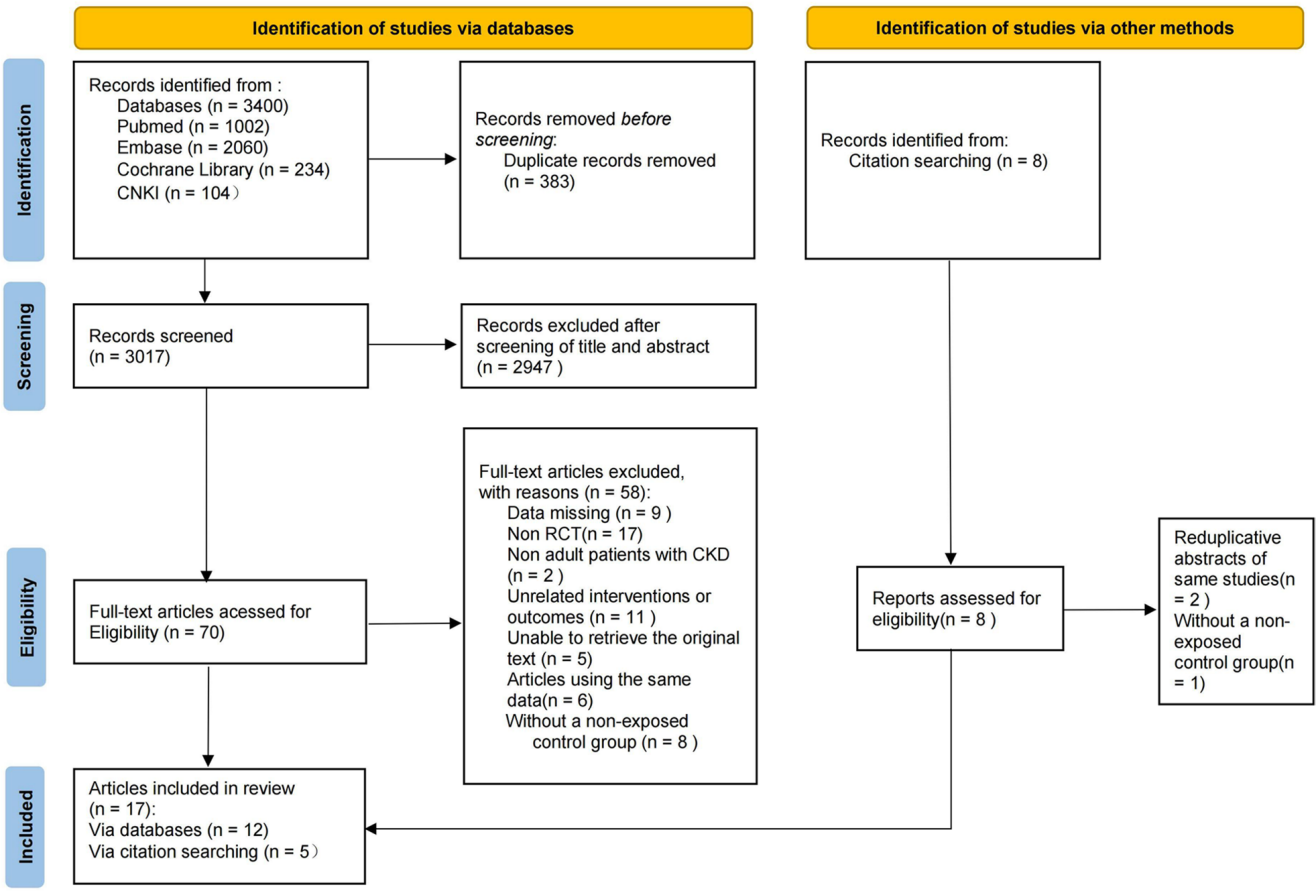
Flow diagram of articles considered for inclusion. Annotation: abbreviation: CNKI, China National Knowledge Infrastructure

Figure 1 shows a flow chart for trial selection. Our initial search yielded 3400 studies; of these, 383 were duplicates and 2947 were ineligible based on our screening of titles and abstracts. Thus, we retrieved full texts of 70 studies. Of these, 17 were not RCT, 9 were data missing, 2 were non adult patients with CKD, 11 were unrelated interventions or outcomes, 5 were unable to retrieve the original text, 6 were using the same data and 8 were without a non-exposed control group. In addition, 8 records were identified from citation searching, in which 2 were duplicates and 1 was without control group. Given significant heterogeneity in SUA level, only trials with SUA ≥ 7.0 mg/dl (420.0 μmol/L) in men or SUA ≥ 6.0 mg/dl (360.0 μmol/L) in women or at least mean baseline SUA ≥ 6.0 mg/dl (360.0 μmol/L) with no prior gout flares were included in this meta-analysis. Doria (2020) [15] and Momeni (2010) [24] were excluded for their baseline SUA level. The inclusion criteria for Doria (2020) [15] is SUA ≥ 267.75 μmol/L. The mean serum level of uric acid was 5.9 ± 1.2 mg/dL and 6.5 ± 2.2 mg/dL respectively in experimental and control groups in the population of the Momeni (2010) [19]. Meanwhile, Tanaka(2020) [25] was excluded because the study population included patients with prior gout. In the Golmohammadi (2017) study [29], as the researchers provided renal function-related data separately for CKD stage 3 and CKD stage 4, without offering data for the overall population, we consider this as two separate sub-studies: Golmohammadi-1(2017) and Golmohammadi-2 (2017), both of which are collectively included in the meta-analysis. Specific data from Mukri (2018) [30], including the lower quartile (Q1) and upper quartile (Q3) of eGFR, were unavailable. The absence of this information made it impossible to convert the reported "Mean (IQR)" (Interquartile Range) into "mean (SD)" (standard deviation). Nevertheless, data concerning acute kidney injury (AKI) from the Mukri (2018) [30] remain available for extraction and utilization in the meta-analysis.

In total, 17 eligible studies with 2032 participants were included in the meta-analysis. Characteristics and demographic data from each of the 17 studies included in our review are listed in Table 1 [11–17, 28–37]. These studies were published between 2006 and 2023 and had sample sizes ranging from 40 to 441. There was no statistically significant difference between the ULT and control groups at baseline in most trails. In the 17 included studies, febuxostat dosage in the treatment group ranged from 10 to 80 mg/day and allopurinol dosage ranged from 100 to 400 mg/day. The control group in most trials was administered placebo or usual therapy or no treatment. Effects of febuxostat or allopurinol was assessed by measuring the changes in levels of SUA and Scr and the changes of eGFR, incidence of doubling of Scr without the requirement of dialysis and incidence of acute kidney injury (AKI).

**Table 1:** Basic characteristics of included studies

Study	Nation	Sample size	population	Diabetes mellitus —no. (%)		Baseline SUA (μmol/L)		SUA after follow-up(μmol/L)	
				I	C	I	C	I	C
Goicoechea (2012)[12]	Spain	113	CKD stage 3–5	20 (36)	22(39)	470.05 ± 124.95	434.35 ± 95.20	360 ± 71.4	446.25 ± 101.15
Badve (2020)[14]	Australia	363	CKD stage 3–4 with UCR ≥ 265 mg/g, eGFR decrease ≥ 3.0 ml/min/1.73m^2^ the preceding year	104 (57)	106 (59)	487.90 ± 107.10	487.9 ± 101.15	320 [95% CI, 300 -330]	490[95% CI, 470—500]
Kimura (2018)[15]	Japan	441	CKD stage 3 with Asymptomatic hyperuricemia	64(29.2)	68 (30.6)	464.10 ± 53.55	464.1 ± 53.55	249.9 [95%CI,238–261.8]	NA
Golmohammadi (2017)[29]	Iran	196	CKD stage 3 with SUA ≥ 360 μmol/L CKD stage 4 with SUA ≥ 360 μmol/L	35 (36.5)	44 (44)	467.67 ± 80.92	458.15 ± 74.97	CKD stage 3 366.52 ± 85.68 CKD stage 4 378.42 ± 79.73	CKD stage 3 418.28 ± 76.16 CKD stage 4 444.46 ± 86.87
Siu (2006)[36]	China	51	Daily proteinuria ≥ 0.5 g and/or an elevated serum creatinine(Cr) ≥ 120 µmol/L	6(24)	7(27)	580.12 ± 70.21	590.24 ± 99.96	349.86 ± 60.01	599.76 ± 99.96
Tan (2011)[32]	China	140	CKD stage 3–4, T2DM, with SUA 420 ~ 600 μmol/L(male),360 ~ 600 μmol/L(female) and daily proteinuria > 0.5 g	72(100)	68(100)	531.23 ± 57.31	511.90 ± 60.32	330.13 ± 37.65	513.46 ± 59.09
Zhou (2009)[13]	China	98	CKD stage 3–4, with SUA > 420 μmol/L(male),SUA > 360 μmol/L(female).proteinuria > 0.5 g	16(31.4)	21(25.5)	420 ± 36	422 ± 50	347 ± 34	419 ± 51
Liu (2007)[37]	China	47	Scr level 120 ~ 400 μmol/L with SUA > 420 μmol/L(male),SUA > 350 μmol/L(female)	NA	NA	579.1 ± 11.7	590.5 ± 15.5	348.7 ± 12.1	598.6 ± 16.7
Jalal (2017)[17]	USA	80	stage 3 CKD on the basis of Modification of Diet in Renal Disease (MDRD) eGFR of 30–59 ml/min per 1.73m^2^, had elevated serum uric acid levels (defined as 7.0 mg/dl for men and 6.0 mg/dl for women)	23 (61)	25 (61)	493.85 ± 83.30	517.65 ± 95.20	Change from baseline according to treatment192.78 ± 80.33	Change from baseline according to treatment2.98 ± 91.63
Deng (2010)[35]	China	61	Scr level 133 ~ 442 μmol/L with SUA 420 ~ 600 μmol/L(male). 360 ~ 600 μmol/L(female)	8(27.6)	13(40.6)	511.48 ± 60. 31	531.47 ± 57.13	329.86 ± 38. 16	513.56 ± 65.74
Lei (2009)[28]	China	57	Scr level 133 ~ 442 μmol/L with SUA > 420 μmol/L(male), > 360 μmol/L(female)	NA	NA	526 ± 86	518 ± 84	392 ± 67	529 ± 72
Shi (2012)[16]	China	40	IgA nephropathy (IgAN), proteinuria between 0.15 and 2.0 g/24 h with serum albumin level > 3.5 g/dl, Scr < 265.2 μmol/L	NA	NA	470.05 ± 65.45	464.10 ± 65.45	339.15 ± 41.65	440.30 ± 89.25
Sircar (2015)[11]	India	93	CKD stages 3–4 with asymptomatic hyperuricemia	20(44)	15(31)	535.5 ± 119.0	487.9 ± 65.45	309.4 ± 89.25	464.10 ± 59.50
Mukri (2018)[30]	Malaysia	93	CKD stage 3–4 patients with diabetic nephropathy and asymptomatic hyperuricemia(> 404 µmol/L or > 6.8 mg/dl)	47(100)	46(100)	539.5 ± 104.0	537.3 ± 70.6	331.6 ± 139.8	538.7 ± 87.1
Shen (2010)[33]	China	52	Scr level 133 ~ 442 μmol/L with SUA > 420 μmol/L(male),SUA > 350 μmol/L(female)	NA	NA	536 ± 82	529 ± 89	384 ± 72	521 ± 78
Wen (2019)[31]	China	38	CKD stage 3 diabetic nephropathy with serum uric acid ≥ 360 μmol/L	18(100)	20(100)	447.5 ± 83.6	423.4 ± 51.2	301.2 ± 46.9	421.1 ± 55.7
Yang (2023)[34]	China	92	patients with CKD stages 3 and 4 and asymptomatic hyperuricemia; SUA level ≥ 6.5 mg/dL	12 (25.5)	8 (17.8)	522.6 ± 103.2	475.2 ± 53.4	337.80 ± 294.6	468.0 ± 297.0

Study	Basline Kidney function (Scr or eGFR)(μmol/L or mL/min/ 1.73 m^2^)		Kidney function after follow-up (Scr or eGFR)(μmol/L or mL/min/ 1.73 m^2^)		Mean age (years)		Therapy		Duration of follow-up	Modified Jadad scores
	I	C	I	C	I	C	I	C		
Goicoechea(2012)[12]	eGFR 40.6 ± 11.3	eGFR 39.5 ± 12.4	eGFR 42.2 ± 13.2	eGFR 35.9 ± 12.3	72.1 ± 7.9	71.4 ± 9.5	Allopurinol 100 mg/d	Usual therapy	24 monthss	4
Badve (2020)[14]	eGFR 31.6 ± 11.7	eGFR 31.9 ± 12.4	Change from baseline according to treatment eGFR slope − 3.33[95% CI, − 4.11 to − 2.55]	Change from baseline according to treatment eGFR slope − 3.23 [95% CI, − 3.98 to − 2.47]	62.3 ± 12.6	62.6 ± 12.9	Allopurinol 100 mg/d	Placebo	26 months	7
Kimura (2018)[15]	eGFR 45.2 ± 9.5	eGFR 44.9 ± 9.7	eGFR 45.1[95%CI,43.7–46.6]	eGFR 44.3[95% CI, 42.8–45.7]	65.4 ± 12.3	65.3 ± 11.8	Febuxostat10-40 mg/d	Placebo	108 weeks	7
Golmohammadi (2017)[29]	CKD stage 3 eGFR 50.37 ± 11.26 CKD stage 4 eGFR 20.84 ± 5.80	CKD stage 3 eGFR 50.38 ± 13.22 CKD stage 4 eGFR 24.57 ± 3.97	CKD stage 3 eGFR 56.82 ± 16.53 CKD stage 4 eGFR 27.32 ± 16.4	CKD stage 3 eGFR 51.99 ± 15.28 CKD stage 4 eGFR27.48 ± 9.85	NA	NA	Allopurinol 100 mg/d	Placebo	12 months	5
Siu (2006)[36]	Scr 144.98 ± 55.69	Scr 164.43 ± 61.00	Scr 175.92 ± 81.33	Scr 255.48 ± 84.87L	47.7 ± 12.9	48.8 ± 16.8	Allopurinol 100 to 300 mg/d	Usual therapy	12 months	4
Tan (2011)[32]	Scr 228.73 ± 84.60	Scr 218.27 ± 85.04	Scr 305.71 ± 140.96	Scr 399.84 ± 189.26	59.3 ± 9.2	58.6 ± 8.3	Allopurinol	No treatment	6 months	3
Zhou (2009)[13]	eGFR 51.1 ± 13.4 Scr 119 ± 27	eGFR 50.8 ± 12.7 Scr 118 ± 23	eGFR 53.8 ± 13.7 Scr 112 ± 22	eGFR 48.0 ± 11.7 Scr 123 ± 25	58.7 ± 8.9	59.3 ± 7.8	Allopurinol 100 to200 mg/d	No treatment	6 months	3
Liu (2007)[37]	Scr 144.8 ± 22.0	Scr 158.4 ± 25.0	Scr 175.7 ± 23.0	Scr 256.3 ± 51.0	45.6 ± 12.5	46.5 ± 13.8	Allopurinol 100 to200 mg/d	No treatment	12 months	3
Jalal (2017)[17]	CKD stage 3 eGFR 41.3 ± 8.9	CKD stage 3 eGFR 42.4 ± 9.6	NA	NA	55.9 ± 13.7	58.9 ± 9.3	allopurinol100 mg	placebo	12 weeks	6
Deng (2010)[35]	Scr 217.72 ± 83. 60	Scr 227.84 ± 87.32	Scr 303.17 ± 139.69	Scr 401.00 ± 182.69	60.0 ± 11.1	58.8 ± 9.4	Allopurinol 100 to 300 mg/d	No treatment	12 months	3
Lei (2009)[28]	Scr 238 ± 71	Scr 242 ± 62	Scr 227 ± 62	Scr 293 ± 55	48.6 ± 10.2	49. 5 ± 9.8	Allopurinol 100 to 200 mg/d	No treatment	12 months	3
Shi (2012)[16]	eGFR 69.5 ± 26.5	eGFR 63.6 ± 27.5	eGF 73.2 ± 34.8	eGFR 68.9 ± 36.6	39.7 ± 10.0	40.1 ± 10.8	Allopurinol 100 to 300 mg/d	Usual therapy	6 months	5
Sircar (2015)[11]	eGFR 31.5 ± 13.6	eGFR 32.6 ± 11.4	eGFR 33.7 ± 16.6	eGFR 28.2 ± 11.5	56.22 ± 10.87	58.42 ± 14.52	Febuxostat 40 mg /d	Placebo	6 months	7
Mukri (2018)[30]	eGFR 26.2 ± 14.3	eGFR 28.2 ± 19.8	eGFR Mean(IQR) 26.3 (15.2)	eGFR Mean(IQR) 27.6 (20.0)	NA	NA	Febuxostat 40 mg /d	No treatment	6 months	4
Shen (2010)[33]	Scr 235 ± 72	Scr 232 ± 62	Scr 238 ± 65	Scr 296 ± 58	47.1 ± 11.8	47.6 ± 12.4	Allopurinol 100 to 200 mg/d	No treatment	12 months	3
Wen (2019)[31]	eGFR 45.3 ± 10.6Scr 172.9 ± 20.1	eGFR 46.8 ± 9.0 Scr 157.7 ± 38.3	eGFR 53.8 ± 9.6 Scr 148.1 ± 30.2	eGFR 42.7 ± 13.4 Scr 170.6 ± 51 .9	58.73 ± 11.50	57.46 ± 10.96	Febuxostat 20–60 mg /d	No treatment	24 weeks	4
Yang (2023)[34]	eGFR 29.9 ± 10.8	eGFR 32.6 ± 8.7	eGFR Change from baseline according to treatment 0.23 ± 5.26 mL/ min/1.73 m^2^ /year	eGFR Change from baseline according to treatment 0.47 ± 4.48 mL/min/1.73 m^2^ /year	57.0 ± 13.6	56.1 ± 13.2	Febuxostat 20–80 mg /d	routine medical care without uric acid lowering agents	12 months	7

*Abbreviations*: *eGFR* estimated glomerular filtration rate(ml/min/1.73m^2^), *CKD* chronic kidney disease, *Scr* Serum creatinine(μmol/L), *SUA* serum urate acid(μmol/L), *UCR* urinary albumin:creatinine ratio; I,intervention; C,control; *T2DM* Type 2Diabetes Mellitus, *T1DM* Type 1Diabetes Mellitus, *IQR* Interquartile Range

### Risk of bias

The methodologic quality of the results was evaluated by the Cochrane Collaboration risk-of-bias (ROB2) tool [26] and modified Jadad [22–24] scale. Eight (47%) of 17 trials were deemed of high quality, seven (42%) of 17 trials were deemed of moderate quality, two of (12%) of 17 trials were deemed of low quality (Supplementary Fig. 1). Six studies had a modified Jadad scale ranged from 1 to 3 which were considered as low quality, 11 trials had a Jadad scale ranged from 4 to 7 which were considered as high quality (Table 1). Four studies [11, 12, 26, 34] analyzed the intentionality of people who lost follow-up. Five randomized controlled trials were double-blind [11, 12, 14, 15]. Two trials [30, 34] were open label study. No crucial deviations from the intended interventions were reported in one of the 17 trials.

### The change of the levels of uric acid

**Fig. 2:**
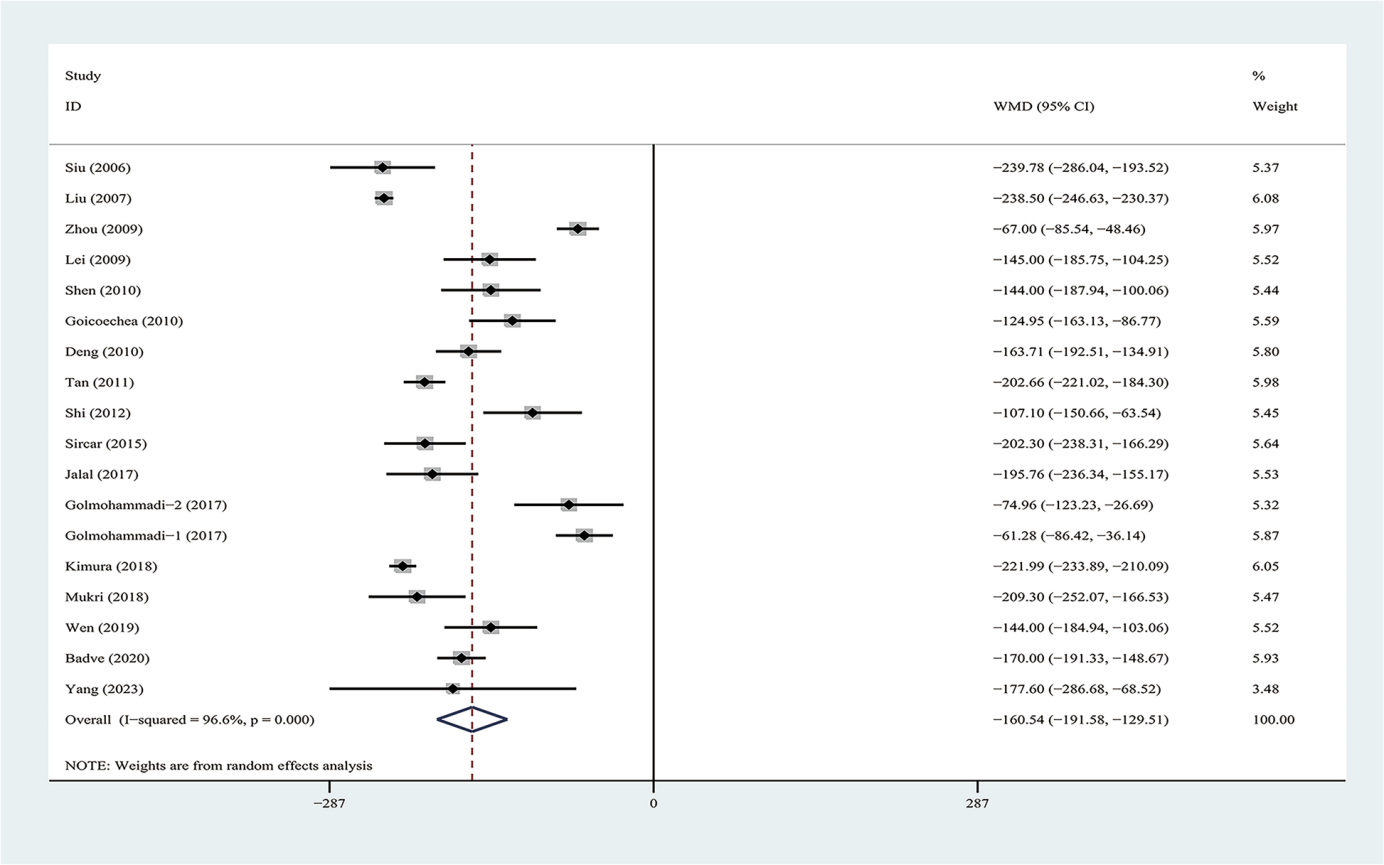
Forest plot for the effect of ULT versus controls on thethe change in the level of uric acid. Annotation: controls, placebo or no treatment; ULT, uric acid-lowering therapy; the Golmohammadi (2017) [29] study were considered as two sub-studies: Golmohammadi-1(2017) and Golmohammadi-2 (2017); data are pooled WMDs with 95% CIs. WMD, Weight Mean differences; CI,confidence interval

The levels of SUA were not significantly different at baseline between treatment and control groups in these 17 studies [11–17, 28–37]. Compared with the control group, ULT group lowered the level of serum uric acid with a weighted mean difference (WMD) of -160.54 μmol/L, 95% CI [-191.58, -129.51] (*p* < 0.001) with significant heterogeneity observed (I^2^ = 96.6%, *p* < 0.001) (Fig. 2). The result of the Egger's test was statistically significant (*p* = 0.022) (Table 2), suggesting a risk of publication bias. However, the sensitivity analysis conducted by excluding individual studies demonstrated a relatively stable result (Supplementary Fig. 2).

**Table 2:** Results of change in uric acid and meta-analysis comparison of uric acid-lowering therapy (ULT) group and control group

Change in uric acid and renal outcome parameters	No of studies	ULT no^a^. / control no^b^	WMD/RR (95%CI)	*p* value	Study heterogeneity				
					Chi-square test	df	I^2^	*p* value	Egger's test *p* value
Changes in uric acid	18	1015/1017	-160.54(-191.58,-129.51)	< 0.001^***^	505.39	17	96.6%	< 0.001^***^	0.022^*^
Changes in eGFR (Length of term)									
Short term	5	162/164	5.74 (2.09, 9.39)	0.002^**^	7.5467	4	47.5%	0.106	0.499
Long term	6	601/604	2.07(0.15,3.98)	0.034^*^	1.3205	5	23.4%	0.259	0.096
Changes in Scr (Length of term)									
Short term	7	229/235	-44.48(-84.03,-4.92)	0.028^*^	1.1e + 03	6	87.7%	< 0.001^***^	0.075
Long term	3	135/129	48.65( -77.30,-20.01)	0.001^**^	1.0e + 03	2	85.1%	< 0.001^***^	0.115
Doubling of Scr without the requirement of dialysis	5	178/177	0.32(0.21, 0.49)	< 0.001^***^	2.36	4	0%	0.653	0.077
Events of AKI	3	274/268	0.97(0.45,2.12)	0.943	1.13	2	0%	0.569	0.638

Control, placebo or no treatment, *ULT* uric acid-lowering therapy; the studies were categorized into three segments based on their follow-up durations: short-term (3–6 months), long-term (> 6 months); the Golmohammadi (2017) [29] study were considered as two sub-studies: Golmohammadi-1(2017) and Golmohammadi-2 (2017); *WMD* Weight Mean differences; *RR* relative risk, *CI* confidence interval, *eGFR* estimated glomerular filtration rate

^*^p < 0.05, 0.001 < p^**^ < 0.05, ***p < 0.001; *Scr* Serum creatinine, *AKI* acute kidney injury

^a^denotes the number of individuals undergoing uric acid-lowering treatment

^b^indicates the number of individuals in the control group

### Primary outcome: the change of eGFR

**Fig. 3:**
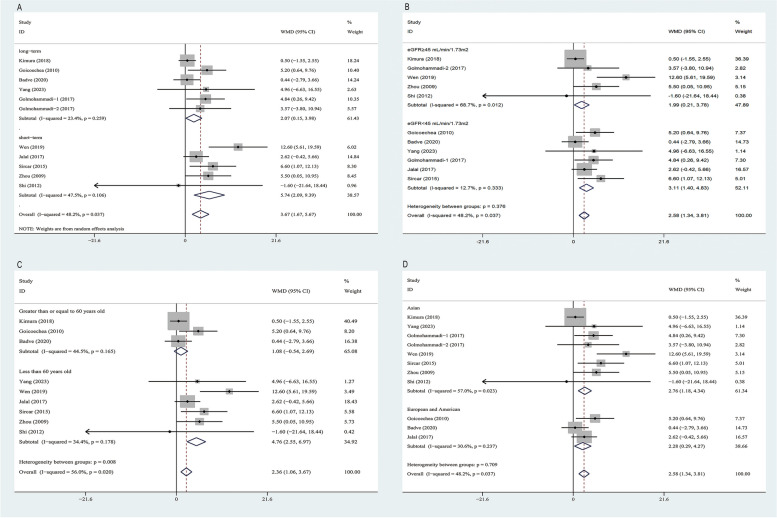
Forest plot for the effect of ULT versus controls on the change in eGFR. **A** Categorized based on follow-up durations, **B** Subgroup-analysis according to renal function (the baseline mean eGFR). **C** Subgroup-analysis according to age. **D** Subgroup-analysis according to the countries of the included trails. Annotation: controls, placebo or no treatment; ULT, uric acid-lowering therapy; the studies were categorized into three segments based on their follow-up durations: short-term (3–6 months), long-term (> 6 months); the Golmohammadi (2017) [29] study were considered as two sub-studies: Golmohammadi-1(2017) and Golmohammadi-2 (2017); data are pooled WMDs with 95% CIs. WMD, Weight Mean differences; CI,confidence interval; eGFR, estimated glomerular filtration rate

Ten RCTs [11–17, 29, 31, 34] involving 1521 participants reported the effects of ULT on the change in eGFR before and after interventions with the average of 12.7 months follow-up period. Overall, compared with the control group, ULT group preserved the loss of estimated eGFR by 3.67 mL/min/1.73m^2^, 95% CI[1.67,5.67], *p* < 0.001 with moderate heterogeneity observed (I^2^ = 48.2%, *p* = 0.037). The studies were^.^categorized into two subgroup based on their follow-up durations: short-term (3–6 months) and long-term (> 6 months). ULT preserved the loss of eGFR at short term (WMD, 5.74 mL/min/1.73m^2^, 95% CI[2.09,9.39]) and long term (2.07 mL/min/1.73m^2^, 95% CI[0.15,3.98]) (Fig. 3A), and the Egger’s test (*p* = 0.499, *p* = 0.096) showed low publication biases (Table 2).

What’s more, we analysed the change of eGFR data stratified by renal function (the baseline mean eGFR), subgroup analyses showed a significant renal benefit from ULT both in patients with the baseline mean eGFR ≥ 45 mL/min/1.73m^2^ (WMD 1.99 mL/min/1.73m^2^, 95% CI [0.21, 3.78] (*p* < 0.001)) and patients with the baseline mean eGFR < 45 mL/min/1.73m^2^ (WMD 2.00 mL/min/1.73m^2^, 95% CI [0.68,3.32] (*p* = 0.003)). The overall test for heterogeneity between two sub-groups was not significant (*p* = 0.376) (Fig. 3B).

We also performed a subgroup analysis by the baseline mean age of the included trails, subgroup analyses showed a significant benefit from ULT in patients with younger than 60 years old(WMD 4.76 mL/min/1.73m^2^, 95% CI [2.60, 7.00] (*p* < 0.001)), no significant heterogeneity was observed (I^2^ = 33.4%, *p* = 0.178), but not for patients more than or equal to 60 years old (WMD 1.08 mL/min/1.73m^2^, 95% CI [-0.54, 2.69] (*p* = 0.192), no significant heterogeneity was observed (I^2^ = 44.5%, *p* = 0.165).The overall test for heterogeneity between two sub-groups was significant (*p* = 0.008) (Fig. 3C).

Finally, we had intended to perform subanalysis by the countries of the included trails, however, there was a significant renal benefit from ULT both in patients from Asian countries (WMD 2.77 mL/min/1.73m^2^, 95% CI[1.19, 4.34] (*p* = 0.001)) and European and American countries (WMD 2.28 mL/min/1.73m^2^, 95% CI [0.29, 4.27] (*p* = 0.025)). The overall test for heterogeneity between two sub-groups showed no significance (*p* = 0.709) (Fig. 3D).

Sensitivity analysis was conducted by excluding individual studies demonstrated a relatively stable result analysis (Supplementary Fig. 3). What's more, when analyzing only the RCTs of high quality (modified Jadad scale > = 4), 9 RCTs [11, 12, 14–17, 29, 31, 34] with high-quality were included in the analysis. There was also a significant renal benefit from ULT (WMD 3.53 mL/min/1.73m^2^, 95% CI [1.40, 5.65] (*p* < 0.001) (Supplementary Fig. 4A). Meanwhile, when deleting low-quality literature (assessed by ROB 2 tool), 8 RCTs [11, 12, 14–17, 29, 34] with high and moderate quality were included in the analysis, there was also a significant renal benefit from ULT (WMD 2.40 mL/min/1.73m^2^, 95% CI [0.85, 3.96] (*p* < 0.001) (Supplementary Fig. 4B).

### Primary outcome: the change of levels of Scr

**Fig. 4:**
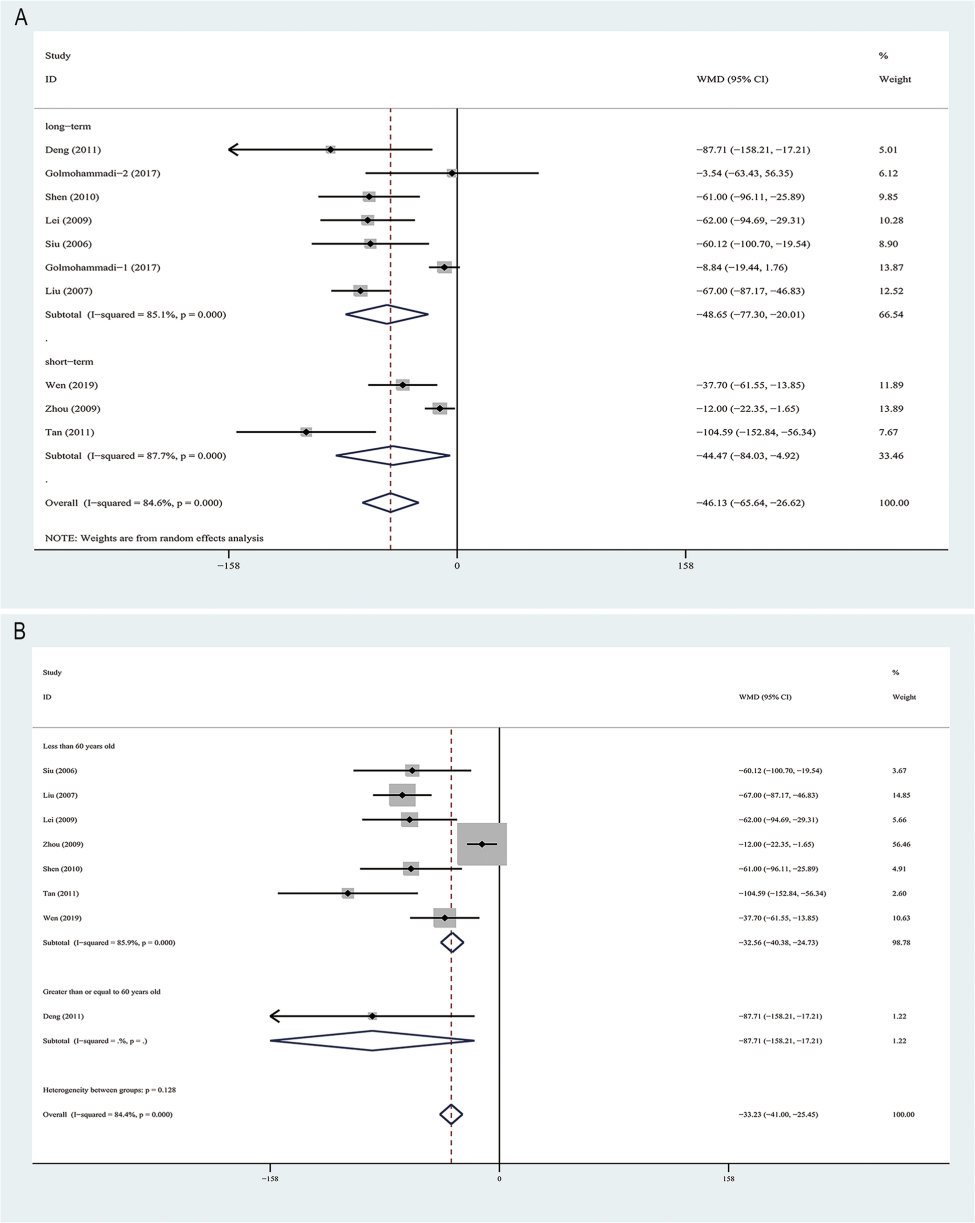
Forest plot for the effect of ULT versus controls on the change in Scr. Annotation: controls, placebo or no treatment; ULT, uric acid-lowering therapy; the studies were categorized into three segments based on their follow-up durations: short-term (3–6 months), long-term (> 6 months); the Golmohammadi (2017) [29] study were considered as two sub-studies: Golmohammadi-1(2017) and Golmohammadi-2 (2017); data are pooled WMDs with 95% CIs. WMD, Weight Mean differences; CI,confidence interval; Scr, serum creatinine

Nine RCTs [13, 28, 29, 31–33, 35–37] evaluated the change of levels of Scr (Fig. 4) in 728 CKD patients with asymptomatic hyperuricemia with the average of 10.2 months follow-up period. Overall, compared with the control group, ULT group reduced the increase of Scr (WMD -46.13, 95% CI [-65.64,-26.62]μmol/L (*p* < 0.001) with significant heterogeneity observed (I^2^ = 84.6%, *p* < 0.001). The study was^.^categorized into two subgroup based on their follow-up duration: short-term (3–6 months) and long-term (> 6 months). ULT reduced the increment of Scr both at short-term (WMD -44.48[-84.03,-4.92]μmol/L) and long-term (WMD -46.13 [-65.64,-26.62]μmol/L) (Fig. 4A). The Egger's test (*p* = 0.075, *p* = 0.115) (Table 2), suggesting low risk of publication biases.

We conducted a subgroup analysis according to whether the baseline mean ages of participants more than or equal 60 or younger than 60 years old and subgroup analyses showed a significant benefit from ULT both in patients younger than 60 years old (WMD -32.55[-40.38,-24.73]μmol/L) (*p* < 0.001) and patients more than or equal to 60 years old (WMD -87.71 [-158.21,-17.21]μmol/L) (*p* = 0.015), the overall test for heterogeneity between two sub-groups showed no significance (*p* = 0.128) (Fig. 4B).

Nine RCTs [13, 28, 29, 31–33, 35–37] evaluated the change of levels of Scr are from Asian countries, so it is impossible to conduct a subgroup analysis between the European and American populations and the Asian populations.

Sensitivity analysis conducted by excluding individual studies one by one demonstrated a relatively stable result analysis (Supplementary Fig. 5). When analyzing with the high quality RCTs [29, 31, 36] (modified Jadad scale > = 4), there was also a significant renal benefit from ULT (WMD -26.91 μmol/L, 95% CI [-51.87, -1.95] (*p* = 0.001) (Supplementary Fig. 6A). When deleting low-quality literature (assessed by ROB 2 tool), there was also a significant renal benefit from ULT (WMD -55.08 mL/min/1.73m^2^, 95% CI [-83.65, -26.52] (*p* < 0.001) (Supplementary Fig. 6B).

### Doubling of serum creatinine(Scr) without the requirement of dialysis

Five RCTs [28, 32, 33, 35, 37] were identified, providing data on the events of doubling of Scr without the requirement of dialysis for 355 patients. There were 22 (22 of 178, 12.4%) and 69 (69 of 177, 39.0%) events of doubling of Scr without the requirement of dialysis in the ULT and control groups, respectively. ULT significantly decreased the incidence of events of doubling of Scr without the requirement of dialysis (relative risk (RR) 32.0%, 95% CI [0.21, 0.49], *p* < 0.001) and no significant heterogeneity was observed (I^2^ = 0%, *p* = 0.653) (Supplementary Fig. 7). The Egger's test (*p* = 0.077) suggesting a low risk of publication bias (Table 2).

### AKI events

Three RCTs [13, 14, 30] were identified with AKI event (Supplementary Fig. 8), including 12 AKI events (12 of 274, 4.4%) in ULT group and 12 (12 of 268, 4.5%) in control group, who developed to AKI. There was no significant difference between the ULT and control groups (RR 97.0%, 95% CI [0.45, 2.12], *p* = 0.943), no significant heterogeneity was observed (I^2^ = 0%, *p* = 0.569). The Egger's test (*p* = 0.638) suggesting a low risk of publication bias (Table 2).

## Discussion

Different countries have differing guidelines for ULT in CKD patients with asymptomatic hyperuricemia in Asian countries, including China and Japan, advocate for ULT, while guidelines in the United States and Europe do not recommend it [3–8]. At the same time, previous studies relating to ULT in CKD patients with asymptomatic hyperuricemia have several limitations as following: firstly, some studies included patients who had prior or acute gouty arthritis. Secondly, the baseline levels of SUA included in the study were controversial and did not reach the criteria of hyperuricemia in some studies [18].

Our study shows that ULT plays an important role in delaying the progression of renal impairment in CKD patients with asymptomatic hyperuricemia, with no significant racial differences according to the subgroup analysis. Due to different guidelines in Asian and non-Asian, further large population RCT studies with high-quality are required to explore whether the benefits of ULT vary among different races.

Furthermore, our study reveals a significant benefit of ULT in both early-stage CKD patients (eGFR > = 45 mL/min/1.73 m^2^) and late-stage CKD patients (eGFR < 45 mL/min/1.73 m^2^). Our study findings are in line with the recommendations of the ACR 2020 guidelines for gout management [20]. According to these guidelines, pharmacologic ULT is recommended for patients with stages 2–5 CKD or end-stage kidney disease (ESKD), particularly those with a history of gout attacks and ongoing hyperuricemia. Our results reinforce these guidelines, demonstrating the efficacy and importance of ULT in these patient groups.

Moreover, the findings of our study indicate that CKD patients with asymptomatic hyperuricemia can benefit from ULT, particularly those younger than 60 years old. This may be due to the fact that many elderly individuals often have multiple comorbidities, such as hypertension and diabetes. A community-based survey in Taiwan by Hsu et al. [38] suggested a weaker correlation between SUA and hypertension in older populations with a longer duration of the disease, indicating uric acid's potential role in younger hypertensive individuals. We speculate that the presence of multiple underlying diseases in elderly patients diminishes the impact of uric acid on renal function. Currently, there is a lack of clinical trials for ULT targeting young patients. Our findings will be instrumental in designing future clinical trials. Further RCT studies with longer follow-up periods are needed to provide more reliable evidence confirming whether ULT has renal protective effects in CKD patients with asymptomatic hyperuricemia.

Compared to previous literature reviews, this meta-analysis included more recent studies, which focused on CKD patients with SUA ≥ 7.0 mg/dl (420.0 μmol/L) in men or SUA ≥ 6.0 mg/dl (360.0 μmol/L) in women or at least mean baseline SUA ≥ 6.0 mg/dl (360.0 μmol/L) with no prior gout flares. The methodological quality of the included literature varied. Overall, the quality of the included randomized controlled trials was relatively high, with 47% being of high quality, 42% of medium quality, and 12% of low quality (Supplementary Fig. 1). Moreover, sensitivity analysis by excluding low-quality studies, also proved the preserved loss of eGFR and reduced the increase of Scr. Statistical heterogeneity (assessed using the Chi-square test and the I^2^ statistic) showed moderate heterogeneity for primary outcomes. To explore the underlying causes of heterogeneity, we carried out subgroup analyses considering various factors such as age, duration of follow-up, baseline eGFR levels, and racial demographics. Egger's test results showed no significant publication bias for primary outcomes (Table 2).

A limitation of this study is that we lack some raw data on the standard deviation of GFR and Scr changes before and after ULT. Some data were calculated by the method recommended by the Cochrane Handbook for Systemic Review and Follmann D’s method [27]. Secondly, this study is based on the analysis of existing clinical research data, and there is considerable heterogeneity between the various RCTs, such as differences in baseline SUA levels, comorbidities, different medications, and so on. Thirdly, due to a lack of a unified and clear definition for the starting level of uric acid reduction and target control, it may potentially affect the results of the study. What’s more, when using Egger's test to examine the relationship between ULT and change in uric acid, there is a certain publication bias in the included literature.

## Conclusion

Our study suggests that uric acid-lowering therapy (ULT) is beneficial in slowing CKD progression in patients with asymptomatic hyperuricemia, both in short-term and long-term follow-ups, and this is consistent across different races and different levels of baseline eGFR. Meanwhile, among patients aged less than 60 years, the protective impact of ULT on renal outcomes is notably enhanced. Nevertheless, it does not show a significant difference in the incidence of AKI. These findings underscore the importance of considering ULT in clinical strategies for CKD patients with asymptomatic hyperuricemia.

### Supplementary Information

**Additional file 1: Supplementary Figure 1.** Assessment of the methodological quality of the included studies. (A) Risk of Bias (B) Risk of Bias Summary.

**Additional file 2: Supplementary Figure 2.** Sensitivity analysis for the of change in uric acid. Annotation: sensitivity analysis was performed by eliminating studies one by one and recalculating the pooled effect.

**Additional file 3: Supplementary Figure 3.** Sensitivity analysis was performed by eliminating studies one by one for the change in eGFR. Annotation: sensitivity analysis was performed by eliminating studies one by one and recalculating the pooled effect; the studies were categorized into three segments based on their follow-up durations: short-term (3-6 months), long-term (>6 months); the Golmohammadi (2017) [29] study were considered as two sub-studies: Golmohammadi-1(2017) and Golmohammadi-2 (2017); data are pooled WMDs with 95% CIs. WMD, Weight Mean differences ; CI,confidence interval; eGFR, estimated glomerular filtration rate.

**Additional file 4: Supplementary Figure 4.** Sensitivity analysis was performed by only including high-quality RCTs for the of change in eGFR. (A) Sensitivity analysis base on high-quality RCTs (assessed by modified Jadad scale). (B) Sensitivity analysis base on high-quality RCTs (assessed by ROB 2 tool). Annotation: the Golmohammadi (2017) [29] study were considered as two sub-studies: Golmohammadi-1(2017) and Golmohammadi-2 (2017);WMD, Weight Mean differences ; RR,relative risk; CI,confidence interval; eGFR, estimated glomerular filtration rate.

**Additional file 5: Supplementary Figure 5.** Sensitivity analysis was performed by eliminating studies one by one for the of change in Serum creatinine (Scr). Annotation: sensitivity analysis was performed by eliminating studies one by one and recalculating the pooled effect.

**Additional file 6: Supplementary Figure 6.** Sensitivity analysis was performed by only including high-quality RCTs for the of change in in Scr. (A) Sensitivity analysis base on high-quality RCTs (assessed by modified Jadad scale). (B) Sensitivity analysis base on high-quality RCTs (assessed by ROB 2 tool). Annotation: the Golmohammadi (2017) [29] study were considered as two sub-studies: Golmohammadi-1(2017) and Golmohammadi-2 (2017);WMD, Weight Mean differences ; RR,relative risk; CI,confidence interval;Scr, Serum creatinine.

**Additional file 7: Supplementary Figure 7.** Forest plot for the effect of ULT versus control on the events of doubling of serum creatinine without the requirement of dialysis. Annotation: control, placebo or no treatment; ULT, uric acid-lowering therapy; the Golmohammadi (2017) [29] study were considered as two sub-studies: Golmohammadi-1(2017) and Golmohammadi-2 (2017); RR,relative risk; CI,confidence interval; AKI, acute kidney injury.

**Additional file 8: Supplementary Figure 8.** Forest plot for the effect of ULT versus control on the events of acute kidney injury (AKI). Annotation: control, placebo or no treatment; ULT, uric acid-lowering therapy; the Golmohammadi (2017) [29] study were considered as two sub-studies: Golmohammadi-1(2017) and Golmohammadi-2 (2017); RR,relative risk; CI,confidence interval; AKI, acute kidney injury.

**Additional file 9. **Search strategy in the PubMed database.

## Data Availability

Data analyzed in this study were a re-analysis of existing data, which are openly available at locations cited in the reference section.
