# Supplementary figures and images for: Effects of uric acid-lowering therapy (ULT) on renal outcomes in CKD patients with asymptomatic hyperuricemia: a systematic review and meta-analysis
Source: BMC Nephrol. 2024 Feb 23;25:63. doi: 10.1186/s12882-024-03491-4 (PMC10893702; doi:10.1186/s12882-024-03491-4)

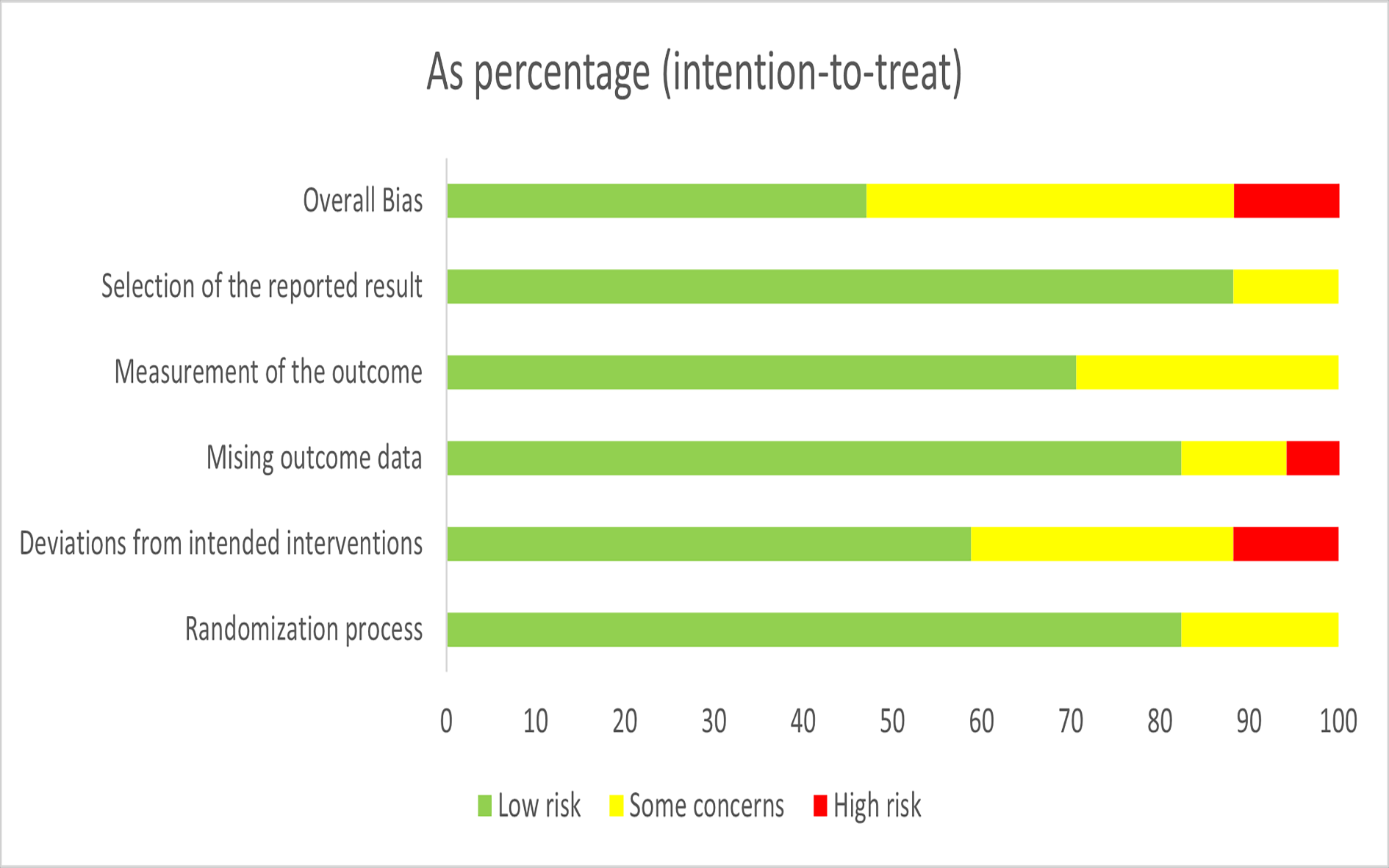

Supplement: Supplementary file 1 — Additional file 1: Supplementary Figure 1. Assessment of the methodological quality of the included studies. (A) Risk of Bias (B) Risk of Bias Summary. [file 12882_2024_3491_MOESM1_ESM.zip › Supplementary Figure 1A.tif]

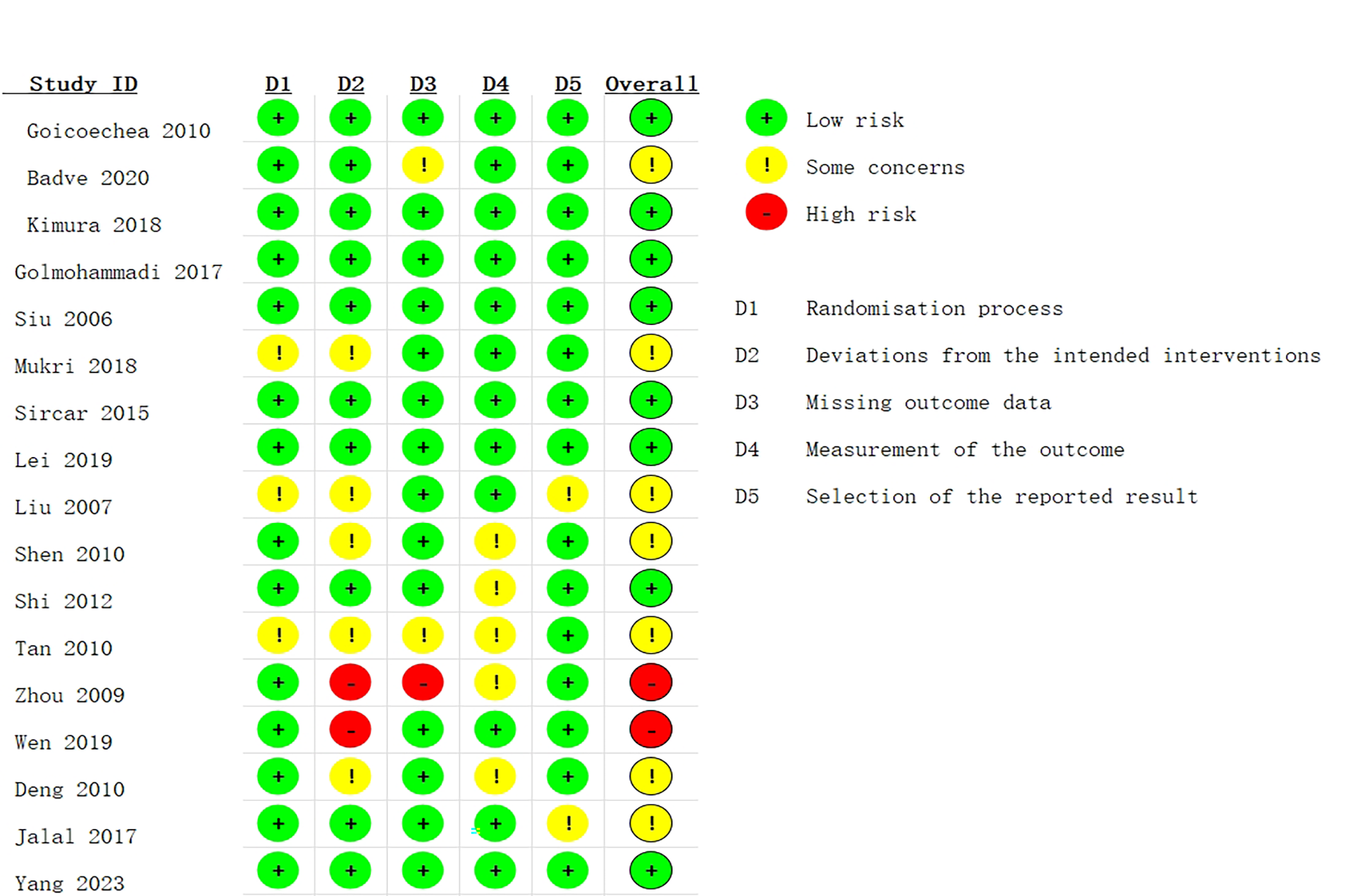

Supplement: Supplementary file 1 — Additional file 1: Supplementary Figure 1. Assessment of the methodological quality of the included studies. (A) Risk of Bias (B) Risk of Bias Summary. [file 12882_2024_3491_MOESM1_ESM.zip › Supplementary Figure 1B.tif]

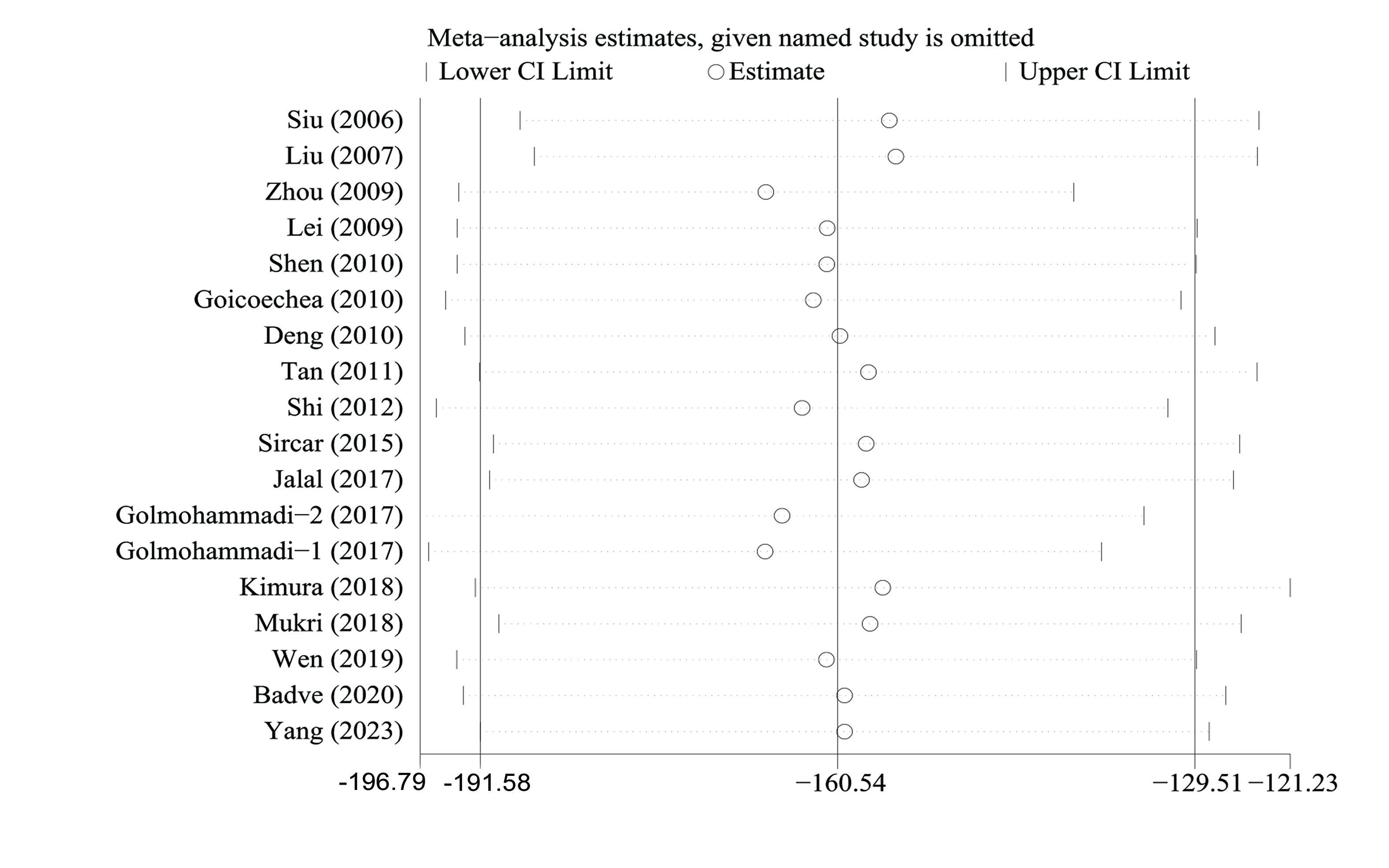

Supplement: Supplementary file 2 — Additional file 2: Supplementary Figure 2. Sensitivity analysis for the of change in uric acid. Annotation: sensitivity analysis was performed by eliminating studies one by one and recalculating the pooled effect. [file 12882_2024_3491_MOESM2_ESM.tif]

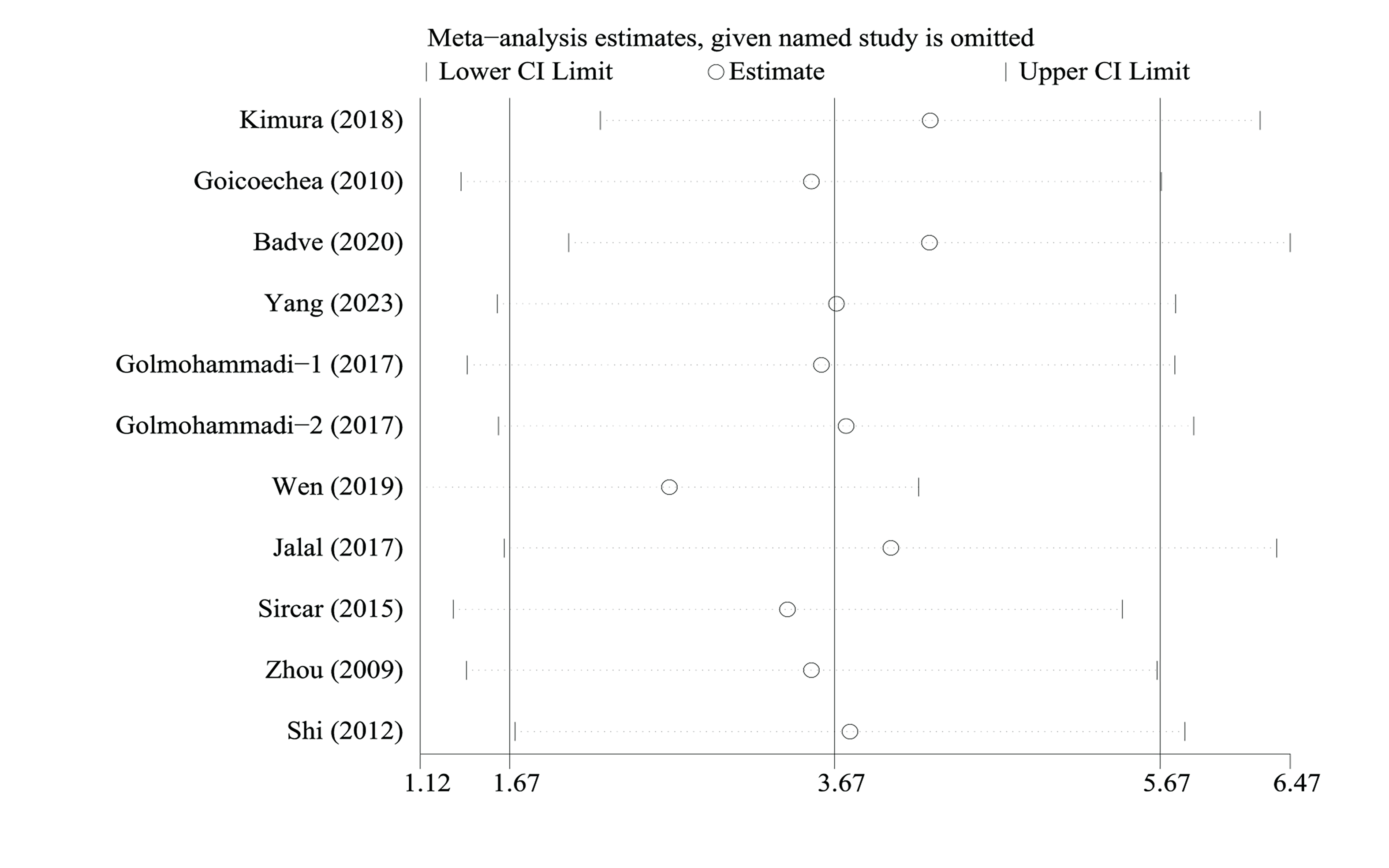

Supplement: Supplementary file 3 — Additional file 3: Supplementary Figure 3. Sensitivity analysis was performed by eliminating studies one by one for the change in eGFR. Annotation: sensitivity analysis was performed by eliminating studies one by one and recalculating the pooled effect; the studies were categorized into three segments based on their follow-up durations: short-term (3-6 months), long-term (>6 months); the Golmohammadi (2017) [29] study were considered as two sub-studies: Golmohammadi-1(2017) and Golmohammadi-2 (2017); data are pooled WMDs with 95% CIs. WMD, Weight Mean differences ; CI,confidence interval; eGFR, estimated glomerular filtration rate. [file 12882_2024_3491_MOESM3_ESM.tif]

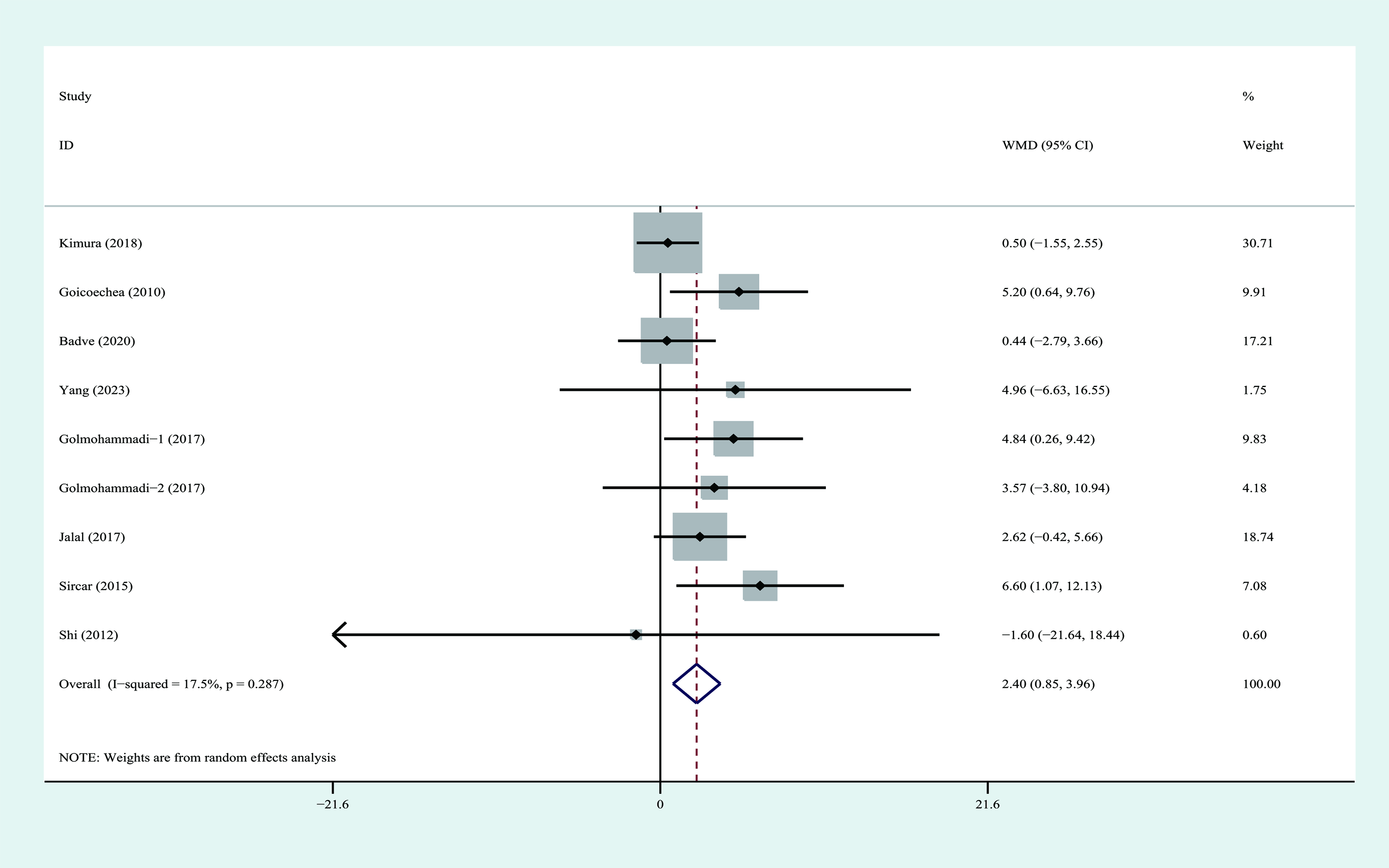

Supplement: Supplementary file 4 — Additional file 4: Supplementary Figure 4. Sensitivity analysis was performed by only including high-quality RCTs for the of change in eGFR. (A) Sensitivity analysis base on high-quality RCTs (assessed by modified Jadad scale). (B) Sensitivity analysis base on high-quality RCTs (assessed by ROB 2 tool). Annotation: the Golmohammadi (2017) [29] study were considered as two sub-studies: Golmohammadi-1(2017) and Golmohammadi-2 (2017);WMD, Weight Mean differences ; RR,relative risk; CI,confidence interval; eGFR, estimated glomerular filtration rate. [file 12882_2024_3491_MOESM4_ESM.zip › Supplementary Figure 4A.tif]

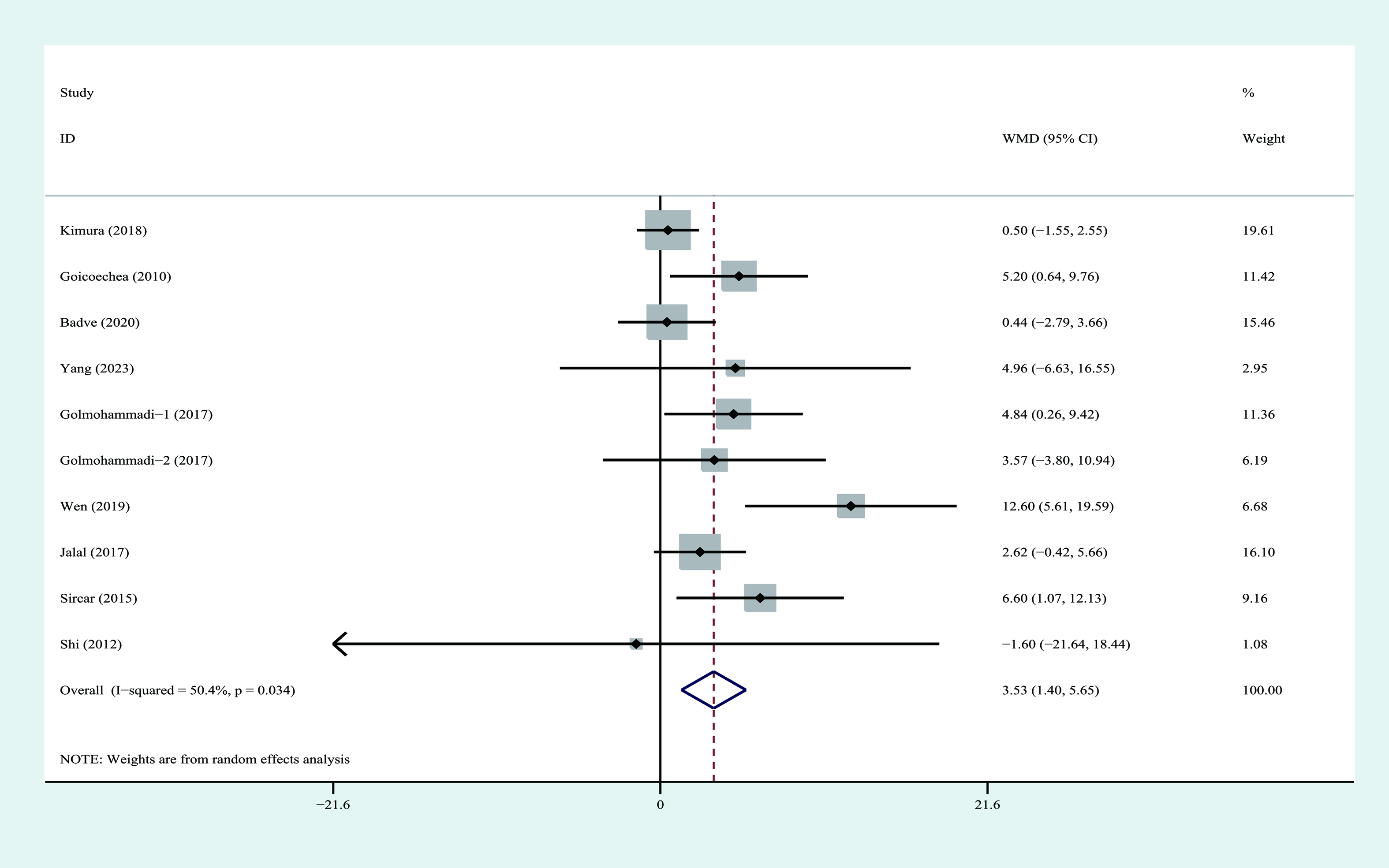

Supplement: Supplementary file 4 — Additional file 4: Supplementary Figure 4. Sensitivity analysis was performed by only including high-quality RCTs for the of change in eGFR. (A) Sensitivity analysis base on high-quality RCTs (assessed by modified Jadad scale). (B) Sensitivity analysis base on high-quality RCTs (assessed by ROB 2 tool). Annotation: the Golmohammadi (2017) [29] study were considered as two sub-studies: Golmohammadi-1(2017) and Golmohammadi-2 (2017);WMD, Weight Mean differences ; RR,relative risk; CI,confidence interval; eGFR, estimated glomerular filtration rate. [file 12882_2024_3491_MOESM4_ESM.zip › Supplementary Figure 4B.tif]

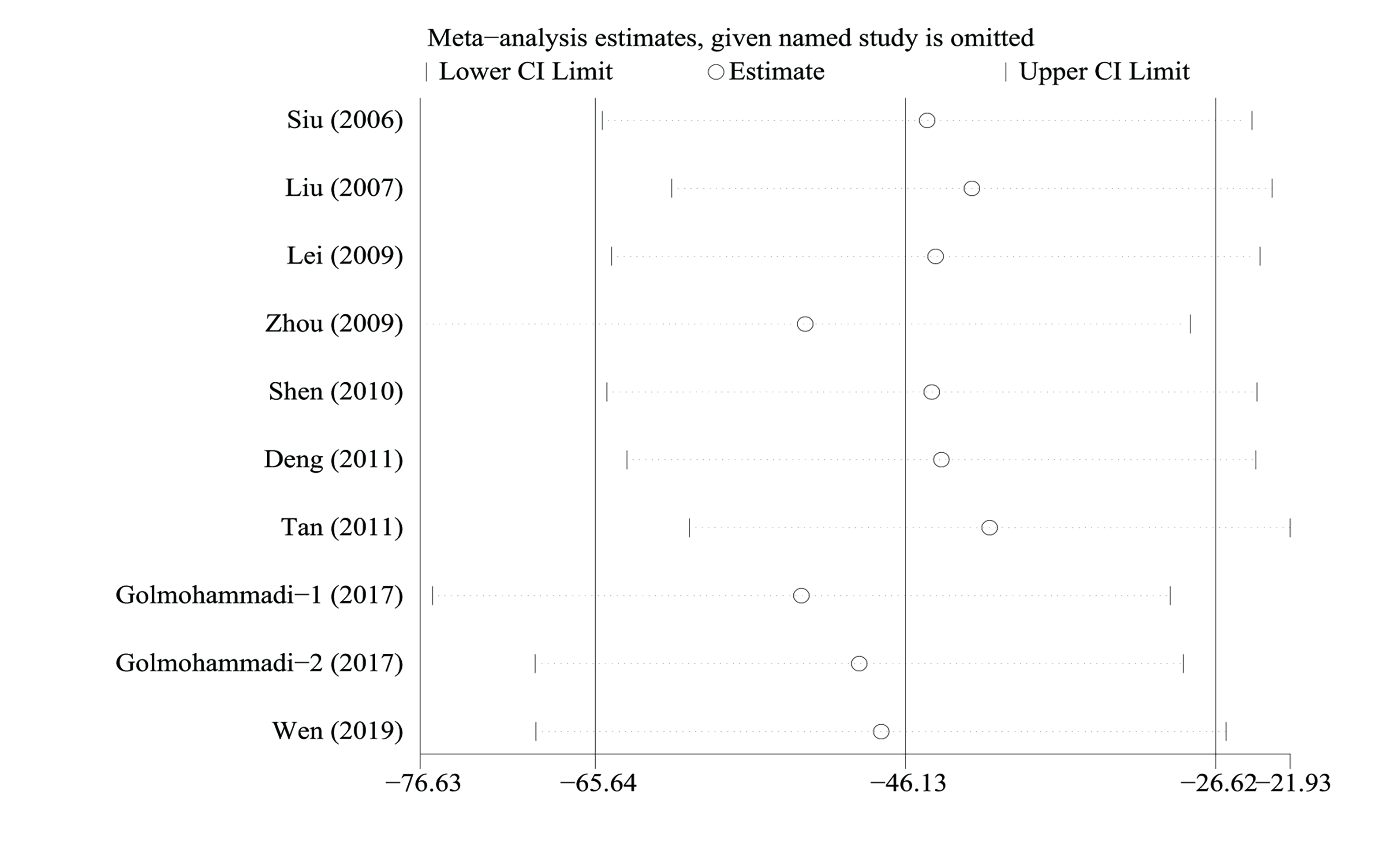

Supplement: Supplementary file 5 — Additional file 5: Supplementary Figure 5. Sensitivity analysis was performed by eliminating studies one by one for the of change in Serum creatinine (Scr). Annotation: sensitivity analysis was performed by eliminating studies one by one and recalculating the pooled effect. [file 12882_2024_3491_MOESM5_ESM.tif]

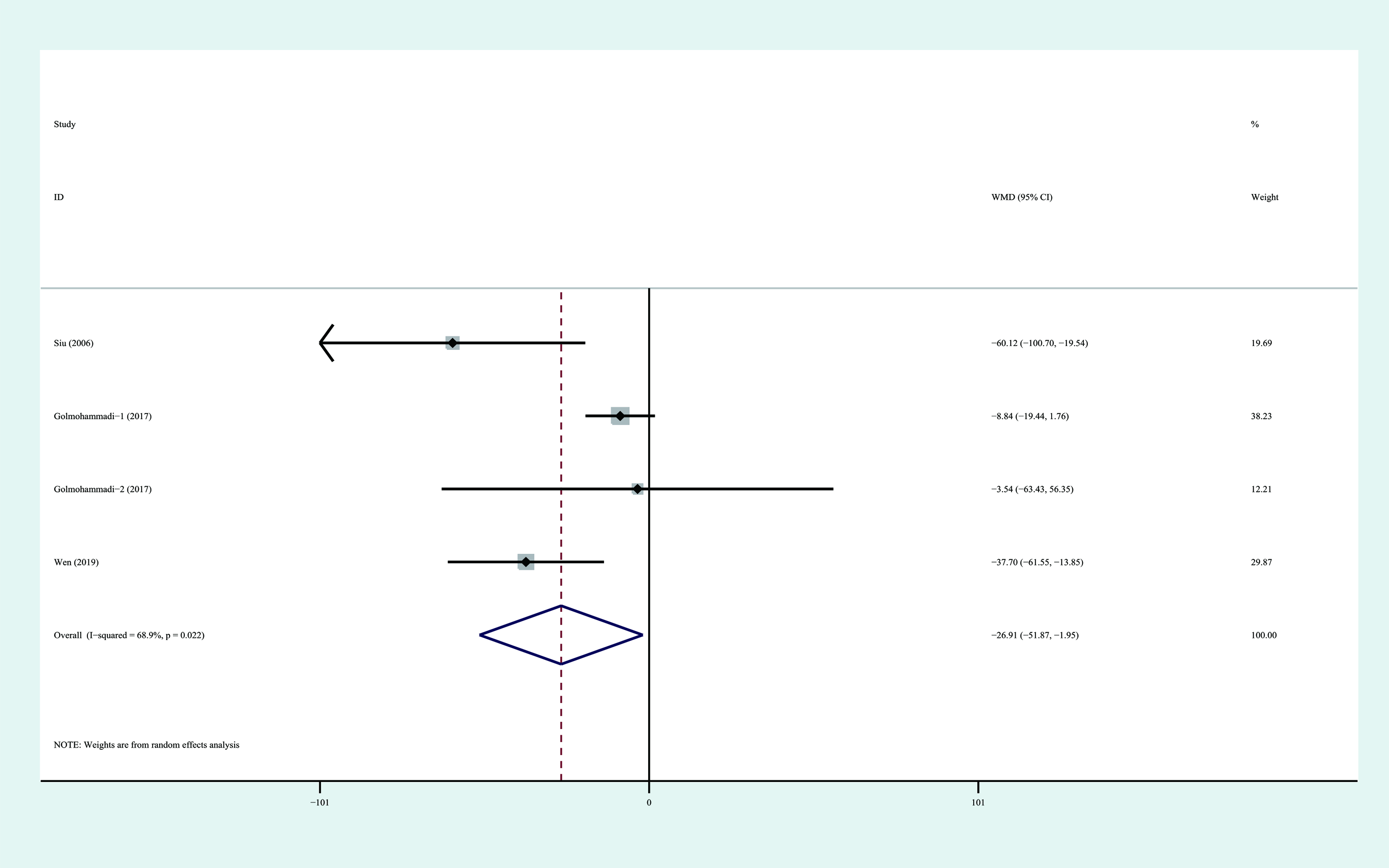

Supplement: Supplementary file 6 — Additional file 6: Supplementary Figure 6. Sensitivity analysis was performed by only including high-quality RCTs for the of change in in Scr. (A) Sensitivity analysis base on high-quality RCTs (assessed by modified Jadad scale). (B) Sensitivity analysis base on high-quality RCTs (assessed by ROB 2 tool). Annotation: the Golmohammadi (2017) [29] study were considered as two sub-studies: Golmohammadi-1(2017) and Golmohammadi-2 (2017);WMD, Weight Mean differences ; RR,relative risk; CI,confidence interval;Scr, Serum creatinine. [file 12882_2024_3491_MOESM6_ESM.zip › Supplementary Figure 6A.tif]

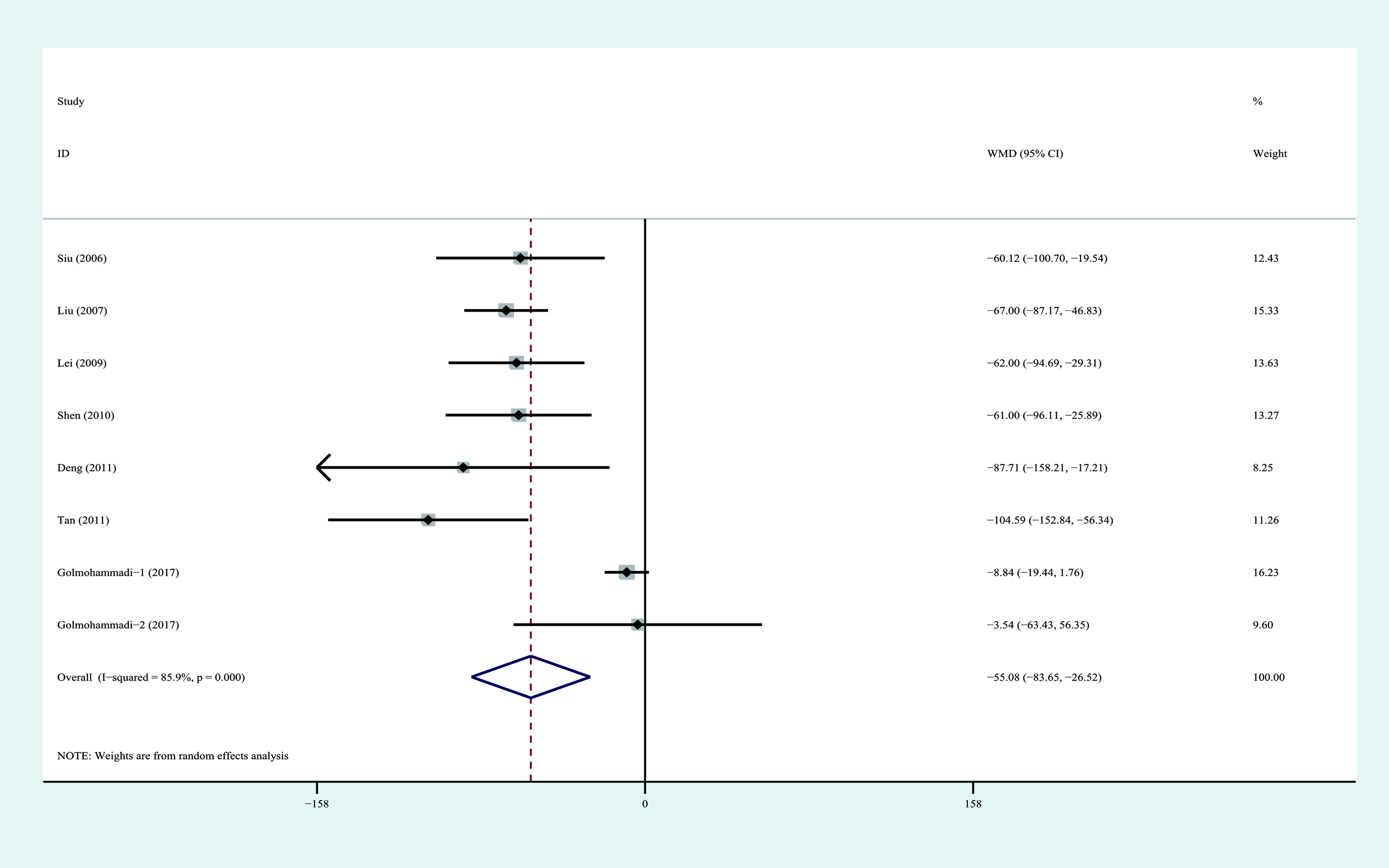

Supplement: Supplementary file 6 — Additional file 6: Supplementary Figure 6. Sensitivity analysis was performed by only including high-quality RCTs for the of change in in Scr. (A) Sensitivity analysis base on high-quality RCTs (assessed by modified Jadad scale). (B) Sensitivity analysis base on high-quality RCTs (assessed by ROB 2 tool). Annotation: the Golmohammadi (2017) [29] study were considered as two sub-studies: Golmohammadi-1(2017) and Golmohammadi-2 (2017);WMD, Weight Mean differences ; RR,relative risk; CI,confidence interval;Scr, Serum creatinine. [file 12882_2024_3491_MOESM6_ESM.zip › Supplementary Figure 6B.tif]

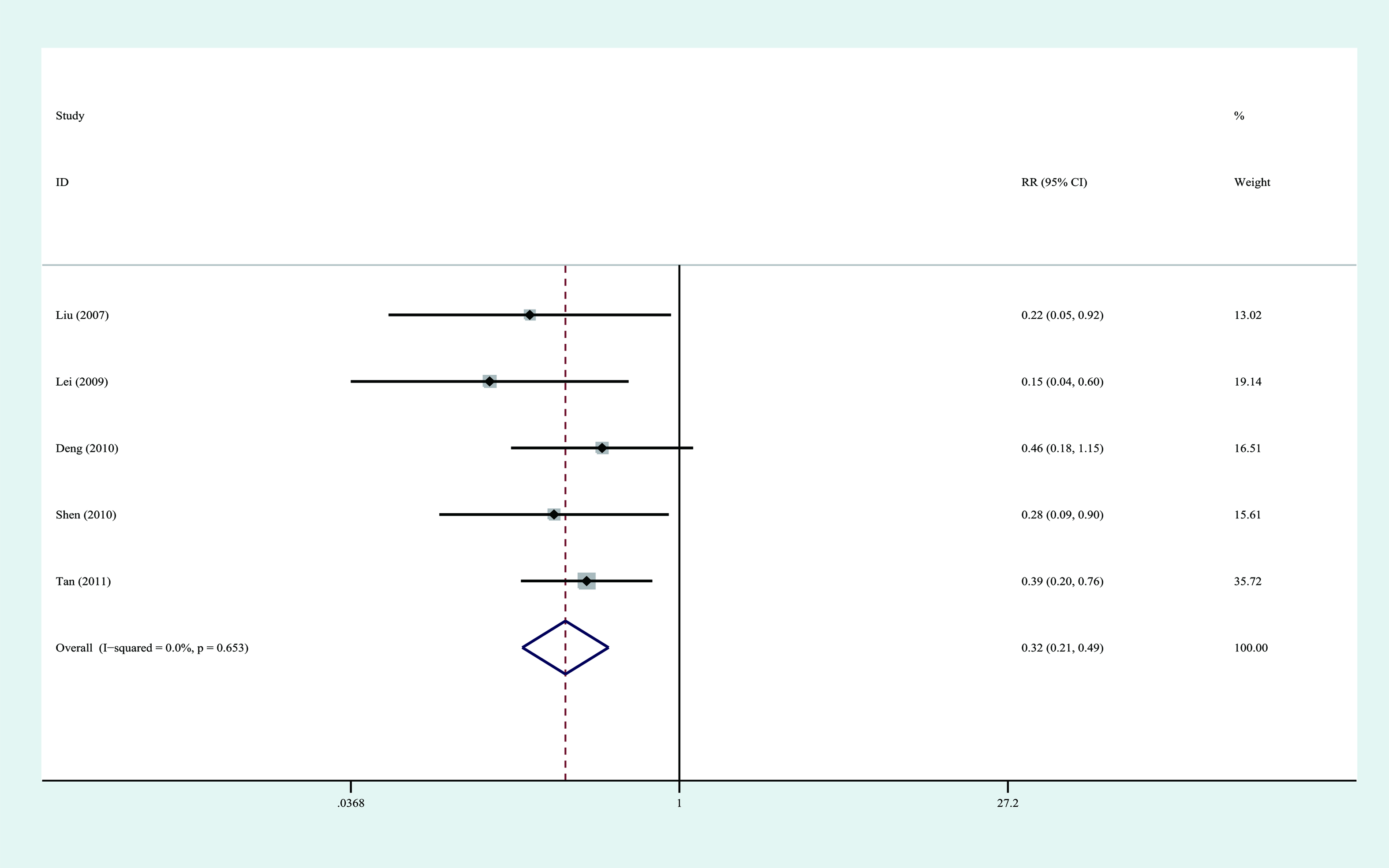

Supplement: Supplementary file 7 — Additional file 7: Supplementary Figure 7. Forest plot for the effect of ULT versus control on the events of doubling of serum creatinine without the requirement of dialysis. Annotation: control, placebo or no treatment; ULT, uric acid-lowering therapy; the Golmohammadi (2017) [29] study were considered as two sub-studies: Golmohammadi-1(2017) and Golmohammadi-2 (2017); RR,relative risk; CI,confidence interval; AKI, acute kidney injury. [file 12882_2024_3491_MOESM7_ESM.tif]

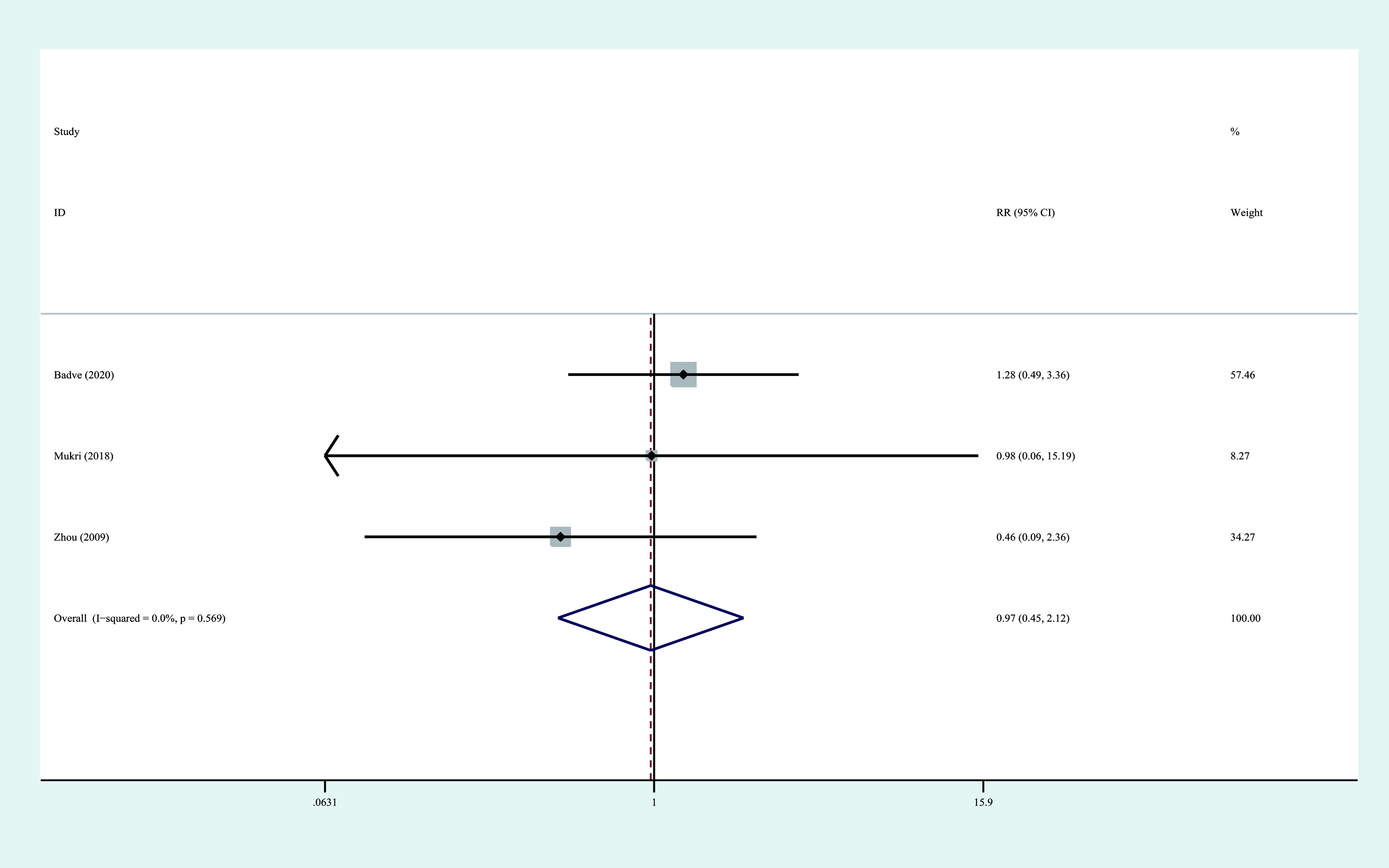

Supplement: Supplementary file 8 — Additional file 8: Supplementary Figure 8. Forest plot for the effect of ULT versus control on the events of acute kidney injury (AKI). Annotation: control, placebo or no treatment; ULT, uric acid-lowering therapy; the Golmohammadi (2017) [29] study were considered as two sub-studies: Golmohammadi-1(2017) and Golmohammadi-2 (2017); RR,relative risk; CI,confidence interval; AKI, acute kidney injury. [file 12882_2024_3491_MOESM8_ESM.tif]
