# Supplementary material for: Effects of uric acid-lowering therapy (ULT) on renal outcomes in CKD patients with asymptomatic hyperuricemia: a systematic review and meta-analysis
Source: BMC Nephrol. 2024 Feb 23;25:63. doi: 10.1186/s12882-024-03491-4 (PMC10893702; doi:10.1186/s12882-024-03491-4)
Supplement: Supplementary file 9 — Additional file 9. Search strategy in the PubMed database. [file 12882_2024_3491_MOESM9_ESM.docx]

Search strategy in the PubMed database:

#1((chronic kidney disease) OR (chronic kidney failure) OR (chronic renal insufficiency) OR (chronic renal failure))

#2((allopurinol) OR (febuxostat) OR (uric acid-lowing therapy) OR (xanthine oxidase) OR(urate-lowering therapy) OR(Benzbromarone) OR(Probenecid) OR(rasburicase) OR(sulfinpyrazone) OR(lesinurad) OR(topiroxostat))

#1 AND #2
